# Precise Dosing of Pramipexole for Low-Dosed Filament Production by Hot Melt Extrusion Applying Various Feeding Methods

**DOI:** 10.3390/pharmaceutics14010216

**Published:** 2022-01-17

**Authors:** Rebecca Chamberlain, Hellen Windolf, Simon Geissler, Julian Quodbach, Jörg Breitkreutz

**Affiliations:** 1 Institute of Pharmaceutics and Biopharmaceutics, Heinrich Heine University, Universitätsstraße 1, 40225 Düsseldorf, Germany; rebecca.chamberlain@hhu.de (R.C.); hellen.windolf@hhu.de (H.W.); joerg.breitkreutz@hhu.de (J.B.); 2 Merck KGaA, Frankfurter Straße 250, 64293 Darmstadt, Germany; simon.geissler@merckgroup.com

**Keywords:** hot melt extrusion, low-dosed filament production, analytics of extruded filaments, fused filament 3D printing, oral dosage form, various dosing techniques, content uniformity, personalized medicine

## Abstract

The aim of this research was the production of low-dosed filaments via hot-melt extrusion (HME) with the model drug pramipexole for the treatment of Parkinson’s disease. The active pharmaceutical ingredient (API) and one of the polymers polyvinyl alcohol (PVA) or basic butylated methacrylate copolymer (bPMMA) were fed by various dosing techniques with the aim of achieving the smallest deviation (RSD) from the target concentration of 0.1% (*w/w*) pramipexole. It was found that deviation from target pramipexole concentration occurred due to degradation products in bPMMA formulations. Additionally, material temperature above 120 °C led to the formation of the anhydrous form of pramipexole within the extruded filaments and need to be considered in the calculation of the recovered API. This study clearly shows that even if equilibrium state of the extrusion parameters was reached, equilibrium condition for drug content was reached relatively late in the process. In addition, the RSD calculated by the Stange–Poole equation was proposed by us to predict the final content uniformity considering the sample size of the analyzed filament. The calculated RSD, depending on sample size and drug load, can serve as upper and lower limits of variation from target concentration and can be used to evaluate the deviations of drug content in equilibrium conditions of the HME process. The lowest deviations from target concentration in equilibrium condition for drug content were obtained in filaments extruded from previously prepared granule mixtures (RSD = 6.00%, acceptance value = 12.2). These promising results can be transferred to other API–excipient combinations to produce low-dosed filaments, which can be used for, e.g., fused filament 3D printing. The introduced calculation of the RSD by Stange–Poole equation can be used for precise determination of the homogeneity of an extruded batch.

## 1. Introduction

High-potent active ingredients have the desired pharmacological effect even at very low concentrations. In this study, the non-ergoline dopamine agonist pramipexole was chosen as a model substance because it is used at low dose strengths (lowest dose < 100 µg) in the treatment of Parkinson’s disease and is water soluble (BCS class I) [1,2]. It is difficult to manufacture low-dosed drug products, wherein the API represents less than 1% (*w/w*) of the total mixture regardless of the number of excipients [3]. Especially for high-potency drugs, which often have a narrow therapeutical window, the API shall be as uniformly distributed within the matrix as possible in order to enable an accurate dosing. The European Pharmacopeia regulates the homogeneity of a batch with the help of the acceptance value for the drug product, which leaves a large margin and is highly dependent on the mass [4]. Therefore, it is recommended that the blend, or in this study, the intermediate filament, be analyzed. Homogeneous mixing of particles with different sizes and shapes is particularly difficult and must be considered in the evaluation of the deviation from target drug content [5,6]. In hot melt extrusion (HME), either blending of powder particles occur before extrusion or mixing takes place alongside extrusion using kneading and conveying elements in the screw configuration. It is also possible that mixing involves both processes, but it must also be remembered that demixing can occur while feeding and/or extruding [7]. Once HME process parameters for producing low-dosed filaments are found, scale-up of HME is much easier to achieve than the scale-up of batch processes, since increasing the batch size requires for this continuous process only a longer run [8]. Since potential drawbacks such as heat stress and shear force on API, which could prevent the production of low-dosed filaments, have been overcome by choosing suitable excipients and self-emulsifying preparations, the focus of hot melt extrusion has increased for the preparation of many drug-loaded extrudates [9,10,11]. After cooling, it is possible that the produced solid dispersion or solid solution shows segregation and re-crystallization, and therefore the maintenance of content uniformity must be tested in a stability study [12]. In previous studies, the API was fed as an anti-solvent suspension into the barrel or even as a physical mixture to form a content uniform batch [13,14]. Sacher et al. developed a novel micro-feeder that was integrated into a continuous manufacturing line with the advantage of a separate feeding of the API [15]. Nevertheless, the properties of the API are indispensable for all described dosing methods in the literature, since in the case of an anti-solvent, the API must not be soluble in the solvent (e.g., water; otherwise, work must be carried out under explosion protection). In the case of a very low concentration of the API in a physical mixture and feeding by a self-made hopper, the solid properties, e.g., flowability, particle size distribution, and adhesion to surfaces, play an important role [16]. In addition, the sample size was not considered in the determination of the drug content, which, however, influences the standard deviation and therefore the validity of the information about content uniformity. The focus of this work was to produce low-dosed filaments for fused filament 3D printing, wherein the active ingredient was homogenously distributed in a solid solution with the help of pharmaceutical-grade polymers. In order for these filaments to be obtained, PVA and bPMMA were chosen to form the polymer matrices. After PVA has been identified as the suitable matrix former, different dosing methods of the API were performed and the findings on the distribution of the low-dosed drug as well as the deviation of the target content (0.1% (*w*/*w*)) were described. Not only were difficulties of the individual dosing steps examined, but also the influence of the process parameters on the starting materials and the API was investigated.

## 2. Materials and Methods

The materials, which have been used for extrusion, are listed in Table 1. As HME is a heat-intensive process, the model substance was chosen regarding thermal resistance. The melting range of PDM, which also represents the temperature of decomposition, is 296–305 °C [1]. PVA was chosen as a polymer, representing an erodible and swelling polymer that predefines prolonged release of the API due to formation of a hydrocolloid matrix [17]. bPMMA was used as a second polymer, depicting an erodible matrix without swelling behavior [18]. For the formulation of printable filaments with bPMMA, mannitol (10% (*w*/*w*)) and sorbitol (5% (*w*/*w*)) were used serving as additives with plasticizing effect. Fumed silica (1% (*w*/*w*)) was used as a glidant for a better flowability of the powder during feeding in the course of dry granulation.

### 2.1. Experimental Procedure of Hot Melt Extrusion Runs

All filaments were prepared by HME with a co-rotating twin-screw extruder with a hot-melt extrusion die (Pharmalab HME 16, Thermo Fisher Scientific, Rockford, IL, USA). A gravimetric feeder (K-SFS-24/6, K-Tron, The Netherlands) was used for all experiments. Data were monitored during extrusion via an in-house written Labview application (Labview 2009 SP 1, National Instruments, Austin, TX, USA) at a frequency of 1 Hz. A vent port was set between kneading zone 1 and 2 for all extrusions except for extrusion run “liquid feeding”. An in-house manufactured die with a diameter of 1.85 mm was used. The desired filament diameter was achieved using a belt hauled-off unit of a winder (Brabender, Duisburg, Germany) with a belt speed of 1.8 m/min, and the filament was pulled through a roll-system with four 360° air flow ring nozzles (Super Air Wipe, Exair, Cincinnati, OH, USA) for active cooling of the melt. With a laser-based diameter measurement module (Laser 2025 T, Sikora, Bremen, Germany), we continuously measured and logged the filament diameter during the process with a readout rate of 1 Hz. If not otherwise stated, the screw speed was set to 30 rpm and powder feed rate was set to 5 g/min. The screw configurations and the temperatures of the heating zones were set for each extrusion and are summarized in Table 2. In this study, the aim was to produce low-dosed filaments in such a way that the API was uniformly distributed and that the target loading of the filaments was 0.1% (*w*/*w*). Polymers (or polymer mixtures with plasticizer) were extruded first without API to adjust the extrusion parameters without wasting API and then the mixture containing the API was added in the same extrusion run [19]. The filaments were cut into 20 cm long filament sticks and sorted chronologically. To determine the content of PDM in the extrudates, the filaments were cut, and random samples were taken from the respective sections. Since the three extrusion setups “split feeding”, “granule feeding”, and “liquid feeding” contained distinctive characteristics, which are herein explained in detail. 

#### 2.1.1. Split Feeding Setup

A second gravimetric feeder (PFW S 20 kg, Three-Tec, Seon, Switzerland) was installed on a table next to the extruder so that the same opening of the barrel could be used for feeders 1 and 2. The feed stock of feeder 1 was only polymer (feed rate: 5 g/min) and a mixture with 15.5% (*w*/*w*) API and 1% (*w*/*w*) anhydrous silica, and polymer was fed with a feed rate of 50 rpm with the help of feeder 2. Two kneading zones were used to guarantee the mixture of polymer and API. The temperature profile was selected the same as for the physical mixture of polyvinyl alcohol and pramipexole (PVA-P).

#### 2.1.2. Liquid Feeding Setup

The feed stock consisting of polymer was fed with the powder feed rate of 2 g/min. A pramipexole solution in demineralized water with a concentration of 40 mg PDM/mL was prepared and was dosed from a 50 mL glass syringe with the help of a syringe pump (Legato 100, KD Scientific, Holliston, MA, USA) with the dosing rate of 50 µL/min through an inhouse-produced barrel element to zone 4. Therefore, the temperature of zones 2–4 was kept low to prevent blockages and guarantee conveyance of the material within the barrel. A vacuum pump was installed to zone 7 to eliminate water and water vapor. To increase the residence time of the melt under the pump so that sufficient degassing would occur, we set the screw speed to 20 rpm [20]. The maximum temperature was reduced to 205 °C.

#### 2.1.3. Granule Feeding Setup

To produce dry granules, we performed two different runs (Table 3) on a roll compactor (BRC 25, L.B. Bohle, Ennigerloh, Germany). Since PVA sticked to the rolls and the ribbons showed lamination, anhydrous silica was added to formulation 2, which was blended for 20 min in a Turbula mixer.

The roll compactor was equipped with a hybrid sealing system, knurled roles, and a 360° rotating conical 1.5 mm rasp sieve (BTS100, L.B. Bohle, Ennigerloh, Germany). The gap width was set to 2 mm, the roll speed to 1 rpm, and the specific compaction force to 2 kN/cm. The fine (<500 µm) was reused for a second compaction to avoid wasting material. Granules were sieved, and the fraction between 500 and 800 µm was used for the extrusion run. The physical mixture of granules with 1% (*w*/*w*) API and PVA granules was feed by feeder 1. The temperature in zone 2 was increased to 120 °C since the torque was too high due to the high granule strength of the material. The screw speed was changed to 20 rpm. The screw configuration included three kneading zones.

### 2.2. Sample Preparation and HPLC Measurements

For sample preparation, filaments were dissolved in flasks (*n* = 1), which were filled with either 0.1 N HCl (bPMMA matrix) or demineralized water (PVA matrix) up to the 100 mL mark. The content of drug loaded filaments was determined by high-performance liquid chromatography (HPLC) analysis. The HPLC system (Dionex, Sunnyvale, CA, USA) was equipped with a quaternary pump (P 580 A, Dionex, Sunnyvale, CA, USA) and an autosampler (ASI-100, Dionex, Sunnyvale, CA, USA). For the HPLC method, a C18-column (Eurospher II 100-5, Knauer, Berlin, Germany) with integrated precolumn was used. The eluent consisted of methanol (mobile phase B) and ammonium acetate buffer (0.05 M, pH 4). The flow rate was set to 1 mL/min, the oven temperature for tempering the column was set to 40 °C, and the injection volume was 50 µL. The gradient was as follows: mobile phase B was increased from 5 to 95% (*v*/*v*) within the first 10 min, it was held for 5 min at 95% (*v*/*v*), and it decreased to 5% (*v*/*v*) again until 20 min after the sample injection. An equilibration time of 3 min per run was allowed to pass before the next sample was injected. Detection was achieved by measuring the UV absorption of the sample at 264 nm.

### 2.3. Production of Melts

The accurate heating of the differential scanning calorimetry device (Mettler Toledo, Giessen, Germany) was used to melt bPMMA and PVA mixtures with different amounts of PDM in aluminum pans (*n* = 1). Therefore, powder mixtures were heated from 20 to 130 °C and were hold for 15, 22.5, and 30 min. Samples were dissolved in 0.1 N HCl and characterized by the HPLC method described in Section 2.2.

### 2.4. Differential Scanning Calorimetry of Pramipexole

Differential scanning calorimetry (DSC, Mettler Toledo, Giessen, Germany) was used to analyze pramipexole dihydrochloride monohydrate. Here, the powder was heated from 20 to 170 °C, cooled down to 20 °C, and heated up again to 320 °C with a heating rate of 10 K/min. The difference of the heat flow between the sample and the standard was detected according to the applied temperature.

### 2.5. Mercury Porosimetry

Mercury porosimetry measurements were performed using mercury porosimetry (Pascal-Quecksilberporosimeter, Thermo Scientific, Waltham, MA, USA). The relationship between intruded volume of mercury and the intrusion pressure was analyzed with the software SOLID version 1.6.6 (Applied Biosystems, Waltham, MA, USA). At the beginning of every determination, the penetrometer was evacuated up to a pressure of 0.013147 MPa. Then, it was filled with mercury under increasing pressure to 0.41 MPa. Penetration pressures were applied in 48 steps in the high-pressure stage. The pore sizes corresponding to the intrusion pressures were calculated by assuming cylindrical pores, a contact angle of 140 °C, and a surface tension for mercury of 485 mN/m. An equilibration time of 10 s was kept for every step between the measurements. The cumulative pore volume was converted into percent for representing a pore size distribution. Porosimeter tests were carried out in duplicate in order to see if the measurement showed any differences.

### 2.6. Laser Diffraction

The particle size distribution of the extruded powders (API and polymer) was determined by laser diffraction using the Mastersizer 3000 (Malvern Panalytical, Malvern, UK) Samples were split using a rotational sample divider (PT100, Retsch, Haan, Germany). They were measured at 0.8-bar dispersion pressure. All off-line samples were measured in threefold.

### 2.7. High-Resolution Mass Spectrometry

The degradation product was separated by UPLC and was analyzed by mass spectrometry (Bruker maXis, Bruker, Billerica, MA, USA) using electrospray ionization (ESI) for fragmentation and measuring in positive mode. For the UPLC method, a C18-column (Waters Atlantis T3 3.0 × 100 mm, 3 µm, Waters, Milford, MA, USA) was used. The eluent consisted of methanol (mobile phase B) and ammonium acetate buffer (0.05 M, pH 4). The flow rate was set to 0.6 mL/min, and the oven temperature for tempering the column was set to 40 °C. The gradient was as follows: mobile phase B was increased from 0 to 20% (*v*/*v*) within the first 4 min, it was held for 3 min at 20% (*v*/*v*), and it decreased to 0% (*v*/*v*) again until 11 min after the sample injection (µL).

### 2.8. Thermogravimetry

Thermal analysis (*n* = 2) was conducted using a thermal analyzer (TG 209 F3 Tarsus^®^, Netzsch, Selb, Germany). The experimental TG sample carrier type S was equipped with a supporting PtRh10-Pt thermocouple to display thermal events during heating in nitrogen atmosphere. The temperature range was set from 30 °C to 300 °C, and the heat rate was 10 K/min.

### 2.9. Theoretical Background of the Deviation from Target Concentration

When the deviation from target concentration of the analyzed concentration of API in hot-melt extruded filaments are assessed, various error sources must be considered. For the analysis of filaments produced by a physical mixture of API and polymer, where only one feeder was used, the deviation from target concentration, expressed as the variance (σ²_total_), can be described with the following Equation [21]:

σ²_total_ = σ²_sampling_ + σ²_analytical_ + σ²_demix_ + σ²_perfect mix_ (1)

The calculation and consideration for each variance can be reconstructed on the basis of the literature sources [21,22,23]. For filaments produced by two feeders, the feeding error resulting from both feeders must be taken into account. Therefore, Equation (1) must be extended by another variance caused by the feeding process, which results in Equation (2):

σ²_total_ = σ²_sampling_ + σ²_analytical_ + σ²_demix_ + σ²_perfect mix_ + σ²_feeding_ (2)

For liquid feeding, the variation of demixing must not be considered if the feeding of an ideal solution is assumed. By adding a second feeder, the error of the gravimetric loss of weight feeding system becomes important for the calculation of the overall deviation from target concentration [24]. Since the analytical error was similar to all extrusion setups, the impact of the remaining errors was analyzed. To find out the lowest deviation of the target concentration, which results from to the (solid) properties of the raw materials and at the same time to consider the sample size, we calculated the relative standard deviation $\left(\mathrm{RSD}\right)$ with the help of a modification of the Stange–Poole equation (Equation (3))

(3) $$\mathrm{RSD}=\frac{100}{x}=\sqrt{\frac{x\;\times \;y\;\times \left({\;\bar{m}}_{x}\times \;y\;+{\;\bar{m}}_{y\;}\times \;x\right)}{M}}$$

where x is the relative mass fraction of pramipexole, y is the relative mass fraction of the polymer, ${\;\bar{m}}_{x}$ is the mean mass of one pramipexole particle, ${\;\bar{m}}_{y}$ is the mean mass of one polymer particle, and M is the mean mass of the filaments [25]. The masses of the powder particles were calculated from the apparent density and of the mean particle size (Dx_50_). This formula was used by Hermes et al. to illustrate that the relative standard deviation of the target concentration of minitablets highly depend on the sample size of powder blends [26]. In this study, the calculation was chosen to assign the deviation from the target concentration to a defined filament weight, which was analyzed for the determination of the content uniformity of the extruded batch. This formula cannot be used in the extrusion setup “liquid feeding” because the API is not particulate but dissolved in water. For the calculation of deviation of the extruded batches, the standard deviation (SD) and the relative standard deviation (RSD) were determined.

## 3. Results 

### 3.1. Pramipexole Degradation within the Hot Melt Extrusion Process

**Figure 1:**
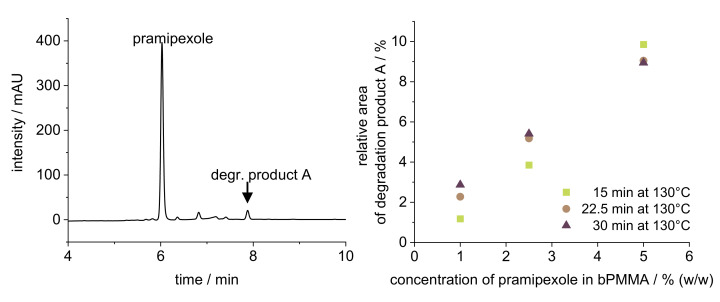
HPLC chromatogram (**left**) of 5% (*w*/*w*) pramipexole filament and evaluation of the relative area degradation product A of the pramipexole peak (**right**) of three different pramipexole concentrations (1, 2.5, and 5% (*w*/*w*)) exposed to 130 °C in DSC for 15, 22.5, and 30 min.

The target drug load of 100% API (drug concentration within the filament: 0.1% (*w*/*w*)) could not be achieved for the physical mixtures of PVA-P and bPMMA-P. One reason for this was the fact that the first extruded API-free polymer amount led to dilution of the afterwards extruded drug-loaded powder mixture in the barrel due to the retention of a dead volume in the barrel, which could not be compensated during the set extrusion time of approximately 60 min. Therefore, after finding suitable extrusion parameters with placebo mixture, we first cleaned the extruder and prepared it again accordingly so that the API was dosed from the beginning and the dilution effect, which would negatively affect the content uniformity, was avoided. Another reason for the low recovery rate of PDM in bPMMA filaments was that degradation products were found in HPLC analysis of filaments containing 5% (*w*/*w*) PDM (Figure 1), which could not be detected in the low-dosed filaments (0.1% (*w*/*w*)) due to the limit of detection of the degradation product. This was further analyzed by preparing melts with 1, 2.5, and 5% (*w*/*w*) PDM according to 2.3 to represent the thermal stress of the extruder but neglecting the shear stress of the HME process. Figure 1 shows that there is a concentration-dependent increase of the degradation product, which is accelerated by thermal stress.

**Figure 2:**
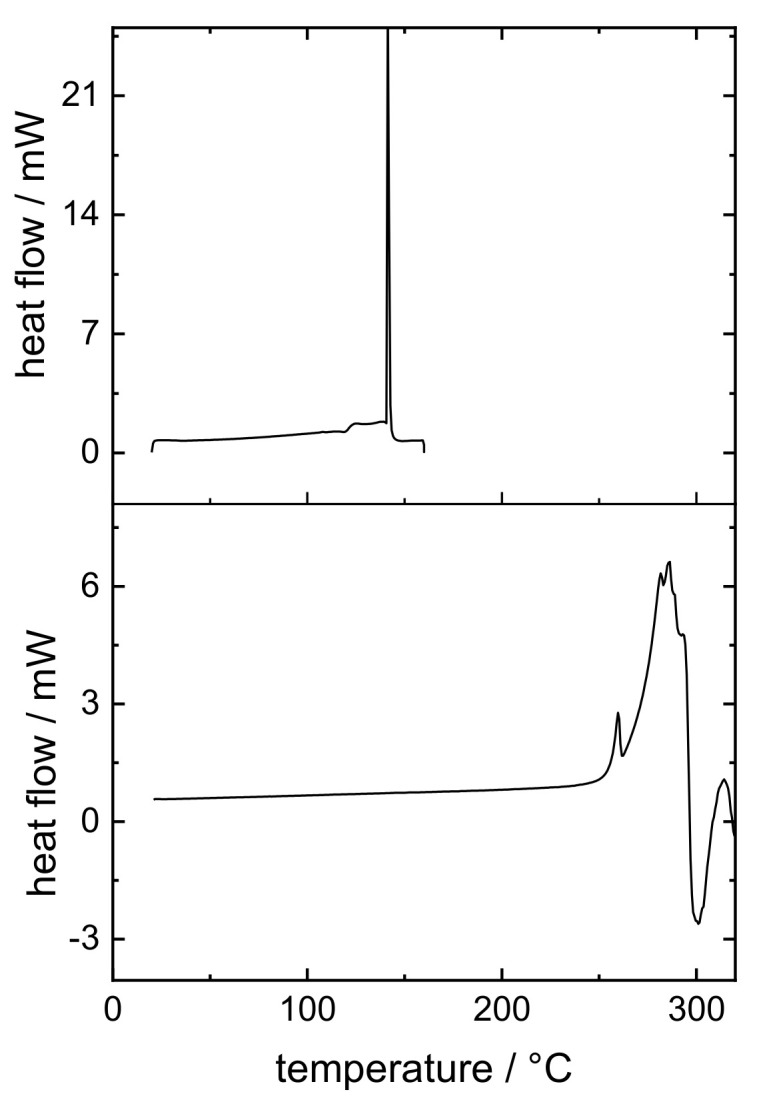
Thermograms of the first and second heating of PDM.

**Figure 3:**
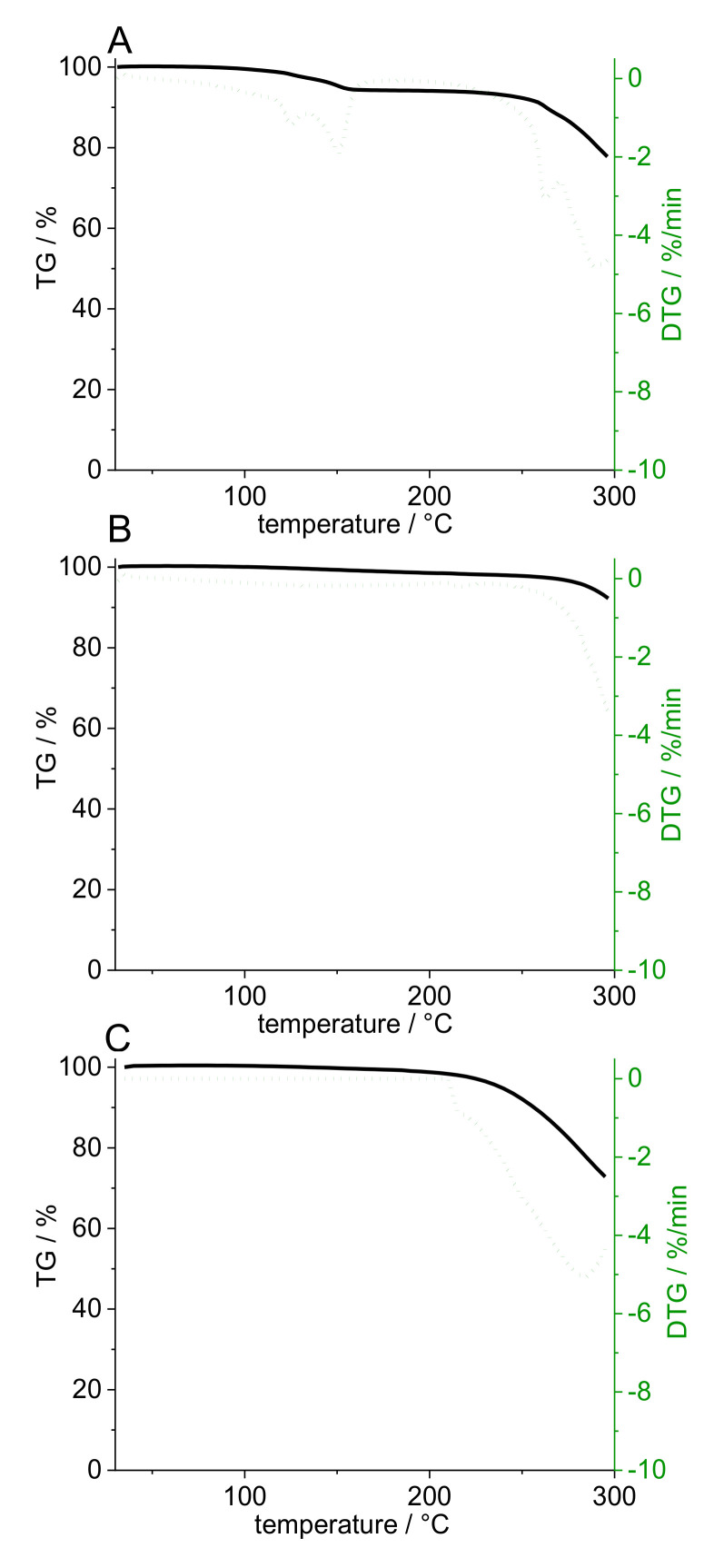
Gravimetric loss (%) and thermal events (%/min) during heating of PDM powder (**A**), PVA placebo filament (**B**), and pramipexole-PVA (5% (*w*/*w*) PDM) filament (**C**).

High-resolution MS was able to identify the degradation product as a pramipexole derivative, an adduct of pramipexole and formaldehyde. Not only 6-propylamino-4,5,6,7-tetrahydrobenzothiazol-2-yl-amine was found, but also 2-methyleneamino-n-propyl-4,5,6,7-tetrahydrobenzothiazol-6-amine. Thus, it was decided that further dosing studies would be conducted with PVA and PDM only, as the degradation product only occurs in combination with bPMMA. Although no degradation product of PDM was found in the PVA filaments in the HPLC chromatograms, a recovery rate of 100% API could not be achieved. Thus, this can be explained by the formation of an anhydrate of PDM due to high temperature used for the extrusion with PVA (up to 220 °C barrel temperature). To confirm this hypothesis, we analyzed the compound by DSC (Figure 2). During initial heating, care was taken to ensure that the degradation temperature, which is also the melting temperature, was not reached. An exothermic event could be detected, which reached its maximum at 140 °C. During second heating, this peak can no longer be detected. It was assumed that during the first heating phase, the crystal structure of PDM decomposed because the water of crystallization was removed, and PDM remained in its anhydrate form. To support this result, we performed an additional thermogravimetric study. Both the starting substance (PDM) and the prepared filaments (PVA filament and filament with 5% (*w*/*w*) PDM in PVA matrix) were examined (Figure 3). It was found that the pure substance of PDM exhibited a weight loss of 5.82% between 100 and 150 °C. This mass difference represents the mass of the hydrate because PDM has a molecular weight of 302 g/mol and the (hydrate) water 18 g/mol, which is about 6% of the salt. The DTG represents the rate of mass loss at given temperature (dm/dT in mg/min unit). Here, the DTG curve indicates a thermal event within the temperature range between 120° and 150 °C with two maxima at 128 °C and 151 °C. While in the TG curve of PVA filament, a decrease in mass of 2.1% could be detected between 100 °C and 240 °C, the mass of 5% PVA-P filaments decreased by 3.44% between 100 °C and 200 °C. For the filaments with PDM, there was a continuous decrease in weight with a small bend in the curve at 170 °C, but no abrupt change in weight between 130 °C and 150 °C, suggesting that the anhydride was present in the filament. Further studies are needed to show why the mass of PVA changed even though the degradation temperature in the literature was above 250 °C. For this study, these results are necessary to calculate the recovery rate. The amount of API was calculated, considering that the anhydrous form rather than the monohydrate was present, which corresponded to a target concentration of 0.094% (*w/w*), not 0.1% (*w/w*). Therefore, the target drug loading was calculated for pramipexole dichloride (PD) and not for pramipexole dihydrochloride monohydrate (PDM). This represents a difference of 6% in the recovery rate and led to the fact that the total amount of API could be found in the equilibrium state for drug content. However, it should be noted that the pharmacopoeia lists only pramipexole dihydrochloride monohydrate [27]. Since a different form of pramipexole is now present due to the extrusion process, it is mandatory that a monography for pramipexole dihydrochloride will be available in the future, as PD is seen as a new active ingredient in clinical studies. This will also occur with other active ingredients, which undergo a similar chemical change due to the hot-melt extrusion process. Therefore, API–polymer combinations must be tested in advance if any degradation product might be formed. Moreover, all active ingredients, which contain hydrate water and are hot-melt extruded at higher temperatures, should be investigated for the presence of the hydrate form.

### 3.2. Equilibrated Conditions for Extrusion

**Figure 4:**
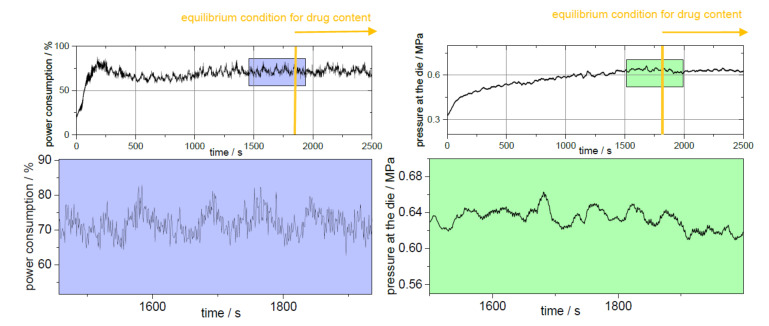
Steady-state conditions of process parameters of extrusion setup “physical mixture” of PVA-P (**left**: power consumption; **right**: pressure at the die) with the time-delayed starting of the equilibrium condition for drug content.

During extrusion, the adjusted process parameters must be kept constant so that a state of equilibrium can occur. The process parameters are responsible for the resulting torque and the pressure at the die [19]. The barrel filling level results not only from the screw speed but also from the dosing rate. Since the barrel filling volume does not have to be the same over the barrel if, for example, kneading elements are installed or the residence time within the barrel varies due to the screw configuration, a true equilibrium is not always achieved. Figure 4 shows the logged process parameters of the extruder for PVA-P “3 kneading zones” for the power consumption and the pressure at the die. For this extrusion run, 25 min passed before both parameters achieved the equilibrium stage. For the other extrusions, the time until equilibrium conditions did not vary much from the shown data (25 ± 5 min). Extrusion run setup with “3 kneading zones” shows a periodically recurring picture of the maxima of the power consumption and the pressure at the die (highlighted by arrows).

**Figure 5:**
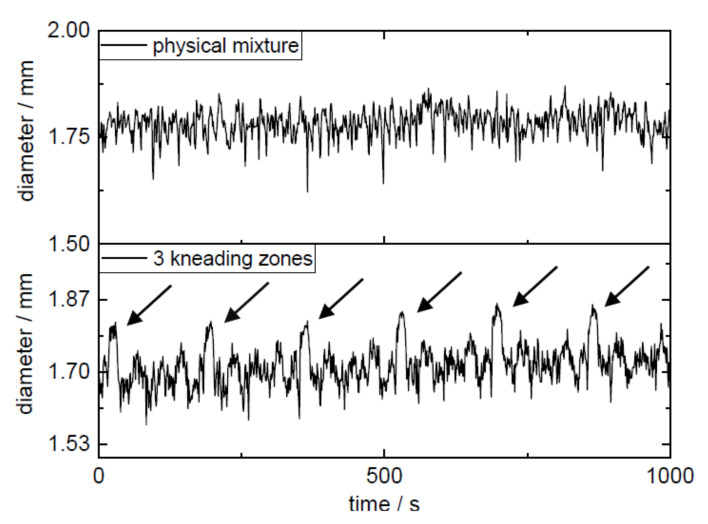
Diameter fluctuation of extrusion setups “physical mixture” and “3 kneading zones”.

The installation of the third kneading zone in the screw configuration resulted in the melt presumably being held up at kneading zone 1 closed to the die, and only when sufficient material accumulated at the 90° kneading elements of the kneading block was the polymer–API melt conveyed further to the nozzle. These periodic fluctuations of the process parameters were reflected in the periodic diameter variations of the filaments (Figure 5, Table 4). Here, every 3 min a maximum diameter was detected, which indicated that the melt was not homogenously transported over the entire time period through the nozzle since the melt accumulated at the additionally introduced kneading zone. The RSD of the target concentration could be improved from 10.76% to 8.28% (AV values of 21.6 and 16.4) by the optimization of the additional kneading capacity (Table 5). The maximum allowed acceptance value for manufactured batches of solid dosage forms (e.g., by fused filament 3D printing) would be exceeded in both cases solely by the inaccuracy of the manufacturing process of the filaments without taking into account the inaccuracy of the printing process. In both cases, demixing (σ²_demix_) might cause the high deviation from the mean concentration (Equation (2)). Therefore, the focus was shifted from the settings of the extruder to the type of dosing and the feeding material, respectively. The extrusion setup “physical mixture” resulted in the highest diameter fluctuation expressed as the interquartile range between 1% and 99% (IQR_1-99_ of 0.152, 0.155, and 0.151 mm), while filaments extruded in extrusion run “liquid feeding” showed the lowest diameter fluctuations with IQR_1-99_ of 0.105, 0.188, and 0.096 mm (Table 4). The filaments extruded with the help of extrusion setting PVA-P “3 kneading zones” achieved the lowest value of 0.017 mm for filament ovality. The highest value for ovality was 0.043 mm, which was obtained by extrusion with granules.

The standard deviation of the target concentration was not improved by dosing the API with a second feeder. Thus, the relative standard deviation RSD of target concentration of API within the filaments of this extrusion run was 11.21% (AV > 27) (Table 5). The installation of a syringe pump, which fed the API solution, led to the RSD of this extrusion run being low (6.35%), but some outliers could be observed, and still the resulting AV value of 14.7 exceeded the maximum limit of L1 (AV = 14). Since the installation of a second feeder or a syringe pump causes the dosing error to originate from both the first and the second dosing system, the dosing error had an additive effect, as Wahl et al. have already demonstrated in their work [28]. This can also be explained by the additional factor in the calculation of the variance (σ²_feeding_) (Equation (3)). The smallest deviations from the target concentration were achieved in extrusion with granules with an RSD of 6.00% and a resulting AV value of 12.2, even though the ovality of the filaments was not satisfactory. For further processing of the filaments into a dosage form by fused-filament 3D printing, round shaped filaments are desirable and diameter fluctuations are undesirable, as they can lead to problems during feeding and variation of the mean diameter can lead to weight fluctuations if the volume flow is kept constant during printing. Diameter fluctuations can be minimized by other methods, e.g., the implementation of a melt pump between barrel and extruder [29], but were not further optimized in this work, since the homogeneity of the drug loading was in focus. By dividing the extrudates into sections during extrusion process, we were able to accurately assign each filament to the extrusion time. Subsequently, the time was examined when the target concentration was reached.

### 3.3. Equilibrium Condition for Drug Content

As already mentioned, hot-melt extrusion was carried out with API from the beginning since it was found that when starting only with polymer, there was an unwanted dilution effect. Even if API was incorporated within the mixture or granule mixture or fed by the second feeder or the syringe pump from the beginning of the extrusion, it can be recognized from the results in Figure 4 that it took a longer period of time to reach the target concentration in the filaments even if a state of equilibrium was established. For “granule feeding” setup, it took 45 min to reach the target concentration of 0.1% (*w*/*w*) PD. Only 30 min passed, when pramipexole was fed in solution (“liquid feeding”), to reach the calculated drug content of 100%. Thus, it is indispensable to use the filaments for further processing only after the time of this adjustment phase. In order for this phase to be detected in the most efficient way, a process analytical technology, which can detect the equilibrium state while extrusion, is desirable. However, the distinction of small quantities (<0.1% (*w*/*w*)) is problematic, and thus the question whether the selected method can analyze the differences must be answered. Wesholowski et al. could not differ between less than 3.51% for carbamazepine and 1.93% for theophylline by using UV–VIS spectroscopy as PAT for concentrations larger than 5% (*w*/*w*) (carbamazepine) and 2.5% (*w*/*w*) (theophylline) [30]. After it was found that an equilibrium state for content uniformity of low-dosed filaments must be set in addition to the extrusion process equilibrium (Figure 4), criteria were needed, which would evaluate the deviations from the target drug content.

### 3.4. Limitation for Deviations of Drug Content

Since no guideline exists for assessing content uniformity of manufacturing intermediates such as drug-loaded filaments, a methodology was developed to define the required sample size depending on variations for the RSD of the content of API [26]. Filaments do not represent dosage forms but serve as intermediates for fused-filament 3D printing [31,32], and therefore the acceptance value will not be used to evaluate the content uniformity of the filaments. Instead, the Stange–Poole equation was used in order to identify the sample size depending relative standard deviation (RSD) from the target drug load. The density of PDM and PVA as well as their particle sizes are shown in Table 6.

**Figure 6:**
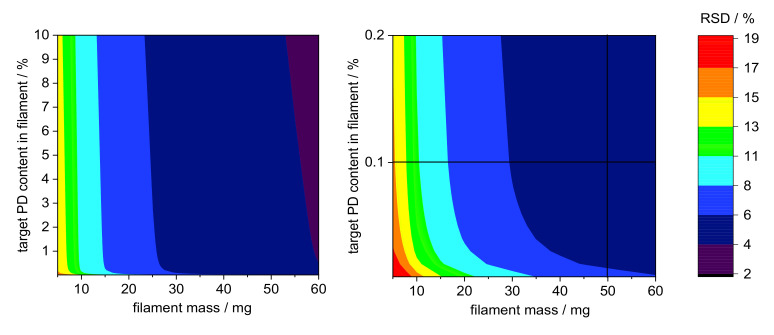
Contour plots for limitations of deviations calculated by Stange–Poole equation: (**left**) colored RSD of PD target concentration depending on target PD content in filament (from 0–10%) and filament mass (0–60 mg); (**right**) RSD from selected filament mass (50 mg) with predetermined PD concentration in filament (0.1% (*w*/*w*)).

**Figure 7:**
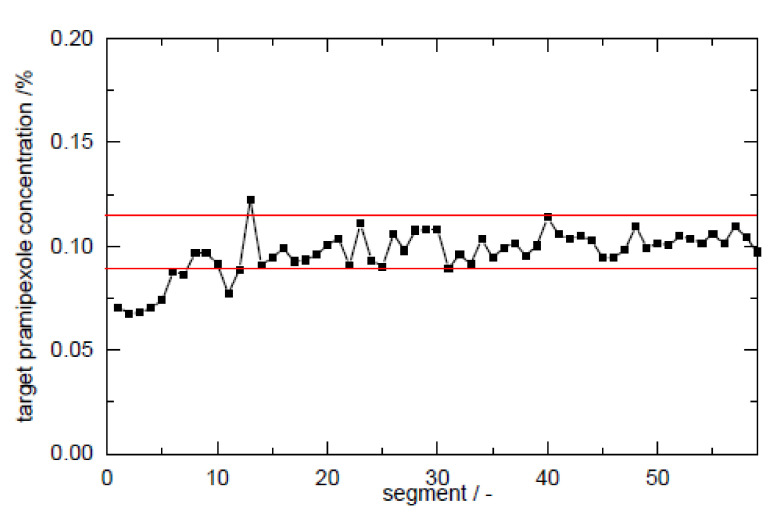
Control chart of extrusion setup “granule feeding” with upper and lower limits calculated from Stange–Poole equation (target content of PDM: 0.1%, filament mass 50 mg).

The calculation of the RSD was performed according to Equation (3) for sample sizes between 5 and 100 mg filament in 0.01% steps for pramipexole content from 0.01 to 10% (*w*/*w*) and is graphical illustrated in a contour plot for sample sizes between 0 and 60 mg (Figure 6). To verify the applicability of the contour plot, we examined higher loaded filaments with respect to the deviation of the content in different sample sizes. As Figure 6 indicates, the RSD decreased with increasing sample size. This was true for both 1 and 5% (*w*/*w*) pramipexole-loaded filaments, which were selected to verify the feasibility of this method (Table 7). The contour plots showed that the standard deviation for the respective sample size was the same for both filaments, although the loading with API varied. For all tested sample sizes, the RSD was within the calculated limits of the Stange–Poole equation or even lower. Thus, for 0.1% (*w*/*w*) loaded filaments, a similarly small RSD had to be maintained. For the determination of content uniformity, the sample size was depicted to be 50 mg, which means that with a drug load of 0.1% (*w*/*w*), the RSD must not exceed 6%. To test whether the limit of ±6% calculated by the modified Stange–Poole equation could be met, we selected the extrusion “granule feeding” to show in equilibrium state for content uniformity whether the deviations were within the allowed range. Since the physical mixture of granules was fed with the help of only one dosing unit, the limits could be complied in equilibrium conditions after filament segment 15 (Figure 7). In equilibrium condition for drug content, 100% of the measured filament fragments were within the set limits (after segment 15).

This pretreatment, namely, the granulation step and the mixing of polymer granules and API-loaded granules before extrusion, is the most promising solution for producing low-dosed filaments with a homogeneous distribution of the API. Even if this extrusion run with pretreatment of the powders seems very complex, it is necessary to go through all these processes, namely, the mixing of powders, the granulation of placebo formulation and API–polymer mixture, the following mixing of the two granules, and the hot melt extrusion process. This setup guaranteed the production of homogeneous low-dosed filaments.

## 4. Discussion

A suitable feeding method was found to obtain the target concentration of 0.1% (*w*/*w*) pramipexole in a pharmaceutical filament that can be used for fused filament 3D printing. With the help of a previously applied dry granulation process, homogeneous pramipexole granules with a drug load of 1% (*w*/*w*) can be produced, which were mixed with granules (1:10 ratio) of the same excipient before extrusion and are fed by the same gravimetrical feeder. Due to the change in the feedstock from powder to granules, however, care must be taken to adjust the extrusion process, especially the temperature and the screw configuration. The largest variations from target drug loading were found for the extrusion runs, wherein the physical mixture was fed by one feeder and where the API was fed with the help of a second gravimetrical feeder (extrusion run “split feeding”) due to demixing processes and the additive variation from the second feeder. In addition, the importance of the consideration of the analyzed sample size was clarified since for the evaluation of the homogeneity of a batch, the sample size should be considered. Accurate calculation of the expected relative standard deviation from target drug load can be made using the Stange–Poole equation. This equation was used to specify a range of how accurately an active ingredient can be dosed at a desired concentration in regard of the sample size. In this study, it was shown that filaments of a mass of 50 mg could be prepared homogeneously and would meet the criteria of content uniformity of the European Pharmacopoeia. Thereby it was important to wait for the accumulation of the active ingredient in the melt in the beginning of the extrusion runs and to take samples in the identified equilibrium condition for drug content. However, this accumulation phase varied, requiring samples to be taken from the beginning to identify the beginning of the equilibrium state. Additionally, it should be tested in future studies as to whether the pre-run of the extrusion might be reduced in time to increase the economic efficiency of the process.

## 5. Conclusions

In this study, we were able to extrude homogenous low-dosed filaments containing PVA and pramipexole, which can be used for fused filament 3D printing to produce solid dosage forms. Since low API concentration causes problems for analytics because the limit of detection for many analytical methods is not reached, it is recommended that higher concentrated filaments be produced first, which are more likely to determine degradation products and changes in solid state properties. Our study showed that with the extrusion with pramipexole and bPMMA, an unwanted degradation product occurred. As well as this, at material temperatures higher than 120 °C, the anhydrous form of PDM was present, and this needed to be considered in the calculation of the drug load. The extrusion of granules with PVA and pramipexole resulted in homogenous filaments after reaching a plateau phase for drug content. Other feeding methods showed higher deviations from target drug content. The results of this study clearly showed that both the dosing error due to segregation during extrusion and the additive error due to an additional feeder had an impact on the homogeneity of the filament. To find limitations for variation of API content, we used the Stange–Poole equation to calculate the allowed RSD. In the plateau phase, the limitations for variation of the drug content could be achieved for most of the samples, which were analyzed by HPLC analysis. In order for the API distribution within the filament to be improved, detailed evaluation of process parameters and adjustment may therefore be required. To evaluate fluctuation in equilibrium state for drug content, one should have inline, non-invasive PAT that can determine quantities of the API, even in very small steps and with less effort than the descriptive HPLC method. In future studies, other low-dosed API–excipient combination will be extruded, and the attempt will be made to reduce the pre-run of the extrusion in time by, e.g., starting with slightly higher concentrations of API. In addition, it will also be investigated as to whether the precision of a pharmaceutical fused filament 3D printer is able to deposit the layers precisely so that higher dosed filaments can be 3D printed to form dosage forms that achieve accurate dosing without the need for the extensive production of low-dosed filaments.

## Figures and Tables

**Table 1:** Extrusion material.

API and Excipients	Function	Manufacturer/Source
Pramipexole 2 HCl·H_2_O (PDM)	API	Chr. Olesen, Gentofte, Denmark
Polyvinyl alcohol (PVA)	matrix	Parteck MXP^®^, Merck, Darmstadt, Germany
Basic butylated methacrylate Copolymer (bPMMA)	matrix	Eudragit E PO^®^, Evonik, Essen, Germany
Fumed silica	glidant	Aerosil^®^ 200 VV Pharma, Evonik, Essen, Germany
Mannitol	plasticizer	Parteck M^®^, Merck, Darmstadt, Germany
Sorbitol	plasticizer	Parteck SI^®^ 200, Merck, Darmstadt, Germany

**Table 2:** Adjusted temperatures during extrusion and screw configurations of performed extrusions.

Temperature Profile in Zone 2–10/°C									
Zone	2	3	4	5	6	7	8	9	10
bPMMA-P (“physical mixture”)	30	100	180	180	180	180	180	195	195
PVA-P (“physical mixture”)	30	180	190	200	220	220	220	220	220
PVA-P (“3 kneading zones”)	30	180	190	200	220	220	220	220	220
PVA-P (“split feeding”)	30	180	190	200	220	220	220	220	220
PVA-P (“granule feeding”)	120	180	190	195	200	200	215	220	220
PVA-P (“liquid feeding”)	20	20	65	170	205	205	205	205	205
Screw Configuration (die–gear) *									
bPMMA-P and PVA-P (“physical mixtures”)	die–10 CE 1 L/D–KZ 1: 5 × 60°–3 × 30°–5 CE 1 L/D–KZ 2: 4 × 90°–5 × 60°–3 × 30°–16 CE 1 L/D–2 CE 3/2 L/D–1 L/D adapter–gear								
PVA-P (“3 kneading zones”)	die–10 CE 1 L/D–KZ 1: 4 × 90°–3 × 60°–3 × 30°–5 CE 1 L/D–KZ 2: 4 × 90°–3 × 60°–3 × 30°–5 CE 1 L/D–KZ 3: 10 × 60°–8 CE 1 L/D–1 CE ½ L/D–2 CE 3/2 L/D–1 L/D adapter–gear								
PVA-P (“split feeding”)	die–8 CE 1 L/D–KZ 1: 4 × 90°–4 × 60°–4 × 30°–5 CE 1 L/D–KZ 2: 5 × 90°–4 × 60°–3 × 30°–6 CE 1 L/D–3 DE L/D–3 CE 1 L/D–4 CE 3/2 L/D–1 L/D adapter–gear								
PVA-P (“granule feeding”)	die–10 CE 1 L/D–KZ 1: 6 × 60°–5 CE 1 L/D–KZ 2: 6 × 60°–6 CE 1 L/D–KZ 3: 4 × 90°–2 × 60°–2 × 30°–10 CE 1 L/D–2 CE 3/2 L/D–1 L/D adapter–gear								
PVA-P (“liquid feeding”)	die–10 CE 1 L/D–KZ 1: 5 × 60°–2 CE 1 L/D–2 CE LP 1 L/D–KZ 2: 5 × 60°–7 CE 1 L/D–KZ 3: 4 × 90°–3 × 60°–3 × 30°–10 CE 1 L/D–2 CE 3/2 L/D–1 L/D adapter–gear								

* CE = conveying element, KZ = kneading zone, DE = distributive element, LP = long pitch.

**Table 3:** Composition of formulations used for roll compaction.

	PVA	Pramipexole Dichloride	Fumed Silica
Formulation 1	100%	-	-
Formulation 2	98%	1%	1%

**Table 4:** Diameter and ovality measurements as inline measurements during extrusion runs expressed as the interquartile range (IQR) between 1 and 99% and the coefficient of variation (CV) in millimeters.

	Diameter		Diameter (x)		Diameter (y)		Ovality (dx–dy)	
Extrusion Setup	IQR_1–99_	CV	IQR_1–99_	CV	IQR_1–99_	CV	IQR_1–99_	CV
bPMMA-P (“physical mixture”)	0.152	1.82	0.155	1.80	0.151	1.85	0.034	6.69
PVA-P (“physical mixture”)	0.582	8.72	0.582	1.57	0.582	8.72	0.022	21.23
PVA-P (“3 kneading zones”)	0.252	2.76	0.256	2.78	0.251	2.75	0.017	14.42
PVA-P (“split feeding”)	0.253	1.77	0.255	1.79	0.253	2.81	0.03	26.72
PVA-P (“granules”)	0.167	2.03	0.179	1.81	0.159	1.99	0.043	23.87
PVA-P (“liquid feeding”)	0.105	1.23	0.118	1.38	0.096	1.24	0.037	15.89

**Table 5:** Content uniformity of the extruded batches: determination of the mean content of pramipexole dihydrochloride, and the standard deviation (SD) from target concentration of 0.1% (*w*/*w*) of pramipexole in PVA filaments and calculation of the relative standard deviation (RSD). The acceptance value was calculated according to Eur. Pharm. 2.9.40 with the acceptability constant of 2.0 at level 2 (*n* = 30).

	Mean Content/%	SD/%	Content Uniformity (RSD)/%	Acceptance Value
PVA-P (“physical mixture”)	0.098	0.011	10.76	21.6
PVA-P (“3 kneading zones”)	0.099	0.008	8.28	16.4
PVA-P (“split feeding”)	0.091	0.010	11.21	27.9
PVA-P (“granules”)	0.101	0.006	6.00	12.2
PVA-P (“liquid feeding”)	0.096	0.006	6.35	14.7

**Table 6:** Mercury density (*n* = 2) and particle sizes of raw materials (*n* = 3).

	PDM	PVA
Density (at 0.4 MPa)	1.2343 g/cm^3^	1.2321 g/cm^3^
Particle size	D_x10_ 6.0 µm D_x50_ 22.2 µm D_x90_ 68.1 µm	D_x10_ 11.8 µm D_x50_ 42.7 µm D_x90_ 96.7 µm

**Table 7:** Determination of the relative standard deviation (RSD) of PVA-P “physical mixture” filaments loaded with 1 and 5% (*w*/*w*) PDM analyzing four different sample sizes (10, 20, 30, and 50 mg filaments) and comparison with results from Stange–Poole equation.

	Filament Mass	RSD	Calculated RSD by Stange–Poole Equation
5% (*w*/*w*) filament	10 mg	8.95%	8–11%
	20 mg	8.00%	6–8%
	30 mg	3.57%	4–6%
	50 mg	4.35%	4–6%
1% (*w*/*w*) filament	10 mg	9.45%	8–11%
	20 mg	5.05%	6–8%
	30 mg	3.19%	4–6%
	50 mg	3.55%	4–6%

## Data Availability

The data presented in this study are available upon request from the corresponding author.

## Abbreviations

API	active pharmaceutical ingredient(s)
AV	acceptance value
bPMMA	basic butylated methacrylate copolymer
CV	coefficient of variation
DSC	differential scanning calorimetry
DTG	derivative thermogravimetry
HME	hot melt extrusion
IQR	interquartile range
MS	mass spectrometry
P	pramipexole
PD	pramipexole dichloride
PDM	pramipexole dichloride monohydrate
PVA	polyvinyl alcohol
RSD	relative standard deviation
SD	standard deviation

## References

[1] Dooley M., Markham A. (1998). Pramipexole. Drugs Aging.

[2] Gültekin H.E., Serdar T., Füsun A. (2019). An effective technology for the development of immediate release solid dosage forms containing low-dose drug: Fused deposition modeling 3D printing. Pharm. Res..

[3] Zheng J. (2009). Formulation and Analytical Development for Low-Dose Oral Drug Products.

[4] European Pharmacopoeia Commission (2020). 2.9.40. Uniformity of dosage units. European Pharmacopoeia.

[5] Ganderton D., Hunter B.M. (1971). A comparison of granules prepared by pan granulation and by massing and screening. J. Pharm. Pharmacol..

[6] Hersey J.A. (1975). Ordered mixing: A new concept in powder mixing practice. Powder Technol..

[7] Tian Y., Jones D.S., Donnelly C., Brannigan T., Li S., Andrews G.P. (2017). A new method of constructing a drug–polymer temperature–composition phase diagram using hot-melt extrusion. Mol. Pharm..

[8] Patil H., Tiwari R.V., Repka M.A. (2016). Hot-melt extrusion: From theory to application in pharmaceutical formulation. AAPS PharmSciTech.

[9] Breitenbach J. (2002). Melt extrusion: From process to drug delivery technology. Eur. J. Pharm. Biopharm..

[10] Loreti G., Maroni A., Dorly del Curto M., Melocchi A., Gazzaniga A., Zema L. (2014). Evaluation of hot-melt extrusion technique in the preparation of HPC matrices for prolonged release. J. Pharm. Sci..

[11] Lagan C., Huckle J.E., Katz J.M., Khorsand B., Daurio D., Andrews G.P., Chung J., Alvarez-Nunez F. (2021). Hot Melt Extrusion of a Thermally Labile, High Melting Point Compound. AAPS PharmSciTech.

[12] Wegiel L.A., Mauer L.J., Edgar K.J., Taylor L.S. (2013). Crystallization of amorphous solid dispersions of resveratrol during preparation and storage—Impact of different polymers. J. Pharm. Sci..

[13] Llusa M., Mohr S., Baumgartner R., Paudel A., Koscher G., Khinast J.G. (2016). Continuous low-dose feeding of highly active pharmaceutical ingredients in hot-melt extrusion. Drug Dev. Ind. Phar..

[14] Park J.B., Kang C.Y., Kang W.S., Choi H.G., Han H.K., Lee B.J. (2013). New investigation of distribution imaging and content uniformity of very low dose drugs using hot-melt extrusion method. Int. J. Pharm..

[15] Sacher S., Heindl N., Urich J.A.A., Kruisz J., Khinast J.G. (2020). A solution for low-dose feeding in continuous pharmaceutical processes. Int. J. Pharm..

[16] Repka M.A., Gerding T.G., Repka S.L., McGinity J.W. (1999). Influence of plasticizers and drugs on the physical-mechanical properties of hydroxypropylcellulose films prepared by hot melt extrusion. Drug Dev. Ind. Phar..

[17] Skowyra J., Pietrzak K., Alhnan M.A. (2015). Fabrication of extended-release patient-tailored prednisolone tablets via fused deposition modelling (FDM) 3D printing. Eur. J. Pharm. Sci..

[18] Wang H., Dumpa N., Bandari S., Durig T., Repka M.A. (2020). Fabrication of Taste-Masked Donut-Shaped Tablets Via Fused Filament Fabrication 3D Printing Paired with Hot-Melt Extrusion Techniques. AAPS PharmSciTech.

[19] Martin C. (2013). Twin screw extrusion for pharmaceutical processes. Melt Extrusion.

[20] Alshahrani S.M., Morott J.T., Alshetaili A.S., Tiwari R.V., Majumdar S., Repka M.A. (2015). Influence of degassing on hot-melt extrusion process. Eur. J. Pharm. Sci..

[21] Gentzler M., Michaels J.N., Tardos G.I. (2015). Quantification of segregation potential for polydisperse, cohesive, multi-component powders and prediction of tablet die-filling performance—A methodology for practical testing, re-formulation and process design. Powder Technol..

[22] Ellison S.L.R., Williams A. (2012). Quantifying Uncertainty in Analytical Measurement.

[23] Anglov T., Byrialsen K., Carstensen J.K., Christensen F., Christensen S., Madsen B.S., Sørensen E., Sørensen J.N., Toftegård K., Winther H. (2003). Uncertainty budget for final assay of a pharmaceutical product based on RP–HPLC. Accredit. Qual. Assur..

[24] Hanson J. (2018). Control of a system of loss-in-weight feeders for drug product continuous manufacturing. Powder Technol..

[25] Egermann H., Frank P. (1992). Novel approach to estimate quality of binary random powder mixtures: Samples of constant volume. I: Derivation of equation. J. Pharm. Sci..

[26] Hermes M. (2012). Kindgerechte, Niedrigdosierte Zubereitungen Mit Enalaprilmaleat. Ph.D. Thesis.

[27] European Pharmacopeia (2014). 8.0, Pramipexole Dihydrochloride Monohydrate. European Pharmacopoeia.

[28] Wahl P.R., Treffer D., Mohr S., Roblegg E., Koscher G., Khinast J.G. (2013). Inline monitoring and a PAT strategy for pharmaceutical hot melt extrusion. Int. J. Pharm..

[29] Peng F., Zhao Z., Xia X., Cakmak M., Vogt B.D. (2018). Enhanced impact resistance of three-dimensional-printed parts with structured filaments. ACS Appl. Mater. Interfaces.

[30] Wesholowski J., Prill S., Berghaus A., Thommes M. (2018). Inline UV/Vis spectroscopy as PAT tool for hot-melt extrusion. Drug Deliv. Transl. Res..

[31] Goyanes A., Buanz A.B., Basit A.W., Gaisford S. (2014). Fused-filament 3D printing (3DP) for fabrication of tablets. Int. J. Pharm..

[32] Windolf H., Chamberlain R., Quodbach J. (2021). Predicting Drug Release from 3D Printed Oral Medicines Based on the Surface Area to Volume Ratio of Tablet Geometry. Pharmaceutics.

